# Low-grade inflammation is negatively associated with physical Health-Related Quality of Life in healthy individuals: Results from The Danish Blood Donor Study (DBDS)

**DOI:** 10.1371/journal.pone.0214468

**Published:** 2019-03-28

**Authors:** Khoa Manh Dinh, Kathrine Agergård Kaspersen, Susan Mikkelsen, Ole Birger Pedersen, Mikkel Steen Petersen, Lise Wegner Thørner, Henrik Hjalgrim, Klaus Rostgaard, Henrik Ullum, Christian Erikstrup

**Affiliations:** 1 Department of Clinical Immunology, Aarhus University Hospital, Aarhus, Denmark; 2 Department of Clinical Immunology, Naestved Hospital, Naestved, Denmark; 3 Department of Clinical Immunology, Copenhagen University Hospital, Rigshospitalet, Copenhagen, Denmark; 4 Epidemiology Research, Statens Serum Institut, Copenhagen, Denmark; 5 Department of Haematology, Copenhagen University Hospital, Rigshospitalet, Copenhagen, Denmark; Universitat de les Illes Balears, SPAIN

## Abstract

**Background:**

Health-Related Quality of Life (HRQL) represent individuals’ subjective assessment of their mental and physical well-being, and is highly predictive of future health. C-reactive protein (CRP) is a well-established marker of inflammation. Low-grade inflammation (LGI), defined as slightly increased CRP levels, is associated with increased risk of several diseases. LGI may reflect subclinical pathology, which could affect individual’s subjective health assessment. This study aimed to examine whether LGI has an independent impact on self-reported health or rather is a mediator of a confounder in a large population of healthy individuals.

**Methods:**

Plasma CRP levels were measured in 17,024 participants from the Danish Blood Donor Study (DBDS). All participants completed a standard questionnaire including smoking status, and the 12-item short-form health survey (SF-12), which is a widely used scale for HRQL. SF-12 is reported as a mental (MCS) and physical (PCS) score. The relationship between LGI (defined as a plasma CRP level between 3 mg/L and 10 mg/L) and MCS or PCS was explored by mediation analysis and adjusted multivariable linear regression analysis. Multiple imputation modelling was used to remedy missing values. The analyses were stratified according to sex and use of combined oral contraception (OC).

**Results:**

In the study, 1,542 (10.3%) participants had LGI. PCS was associated with LGI in all strata, i.e. women using OC: RC = -0.36 points lower PCS in participants with LGI vs no LGI, CI: -0.94 to -0.19, women not using OC: RC = -0.63, CI: -1.05 to -0.21 and men: RC = -0.76, CI: -1.10 to -0.42. But LGI had no impact on MCS. Predictors of lower PCS included obesity, current smoking, and waist circumference in all strata. Physical activity in leisure time was the only factor positively associated with PCS. Age and physical activity in leisure time was associated with increased MCS in all strata whereas current smoking was the only strong predictor of a reduction in MCS. Only a small effect of smoking on PCS was mediated through LGI.

**Conclusion:**

In this population of healthy individuals, LGI had independent impact on lower self-rated physical health score in HRQL in both sexes, but was not associated with self-rated mental health score. A small and significant effect of smoking on physical health score was mediated through LGI.

## Introduction

C-reactive protein (CRP) is an acute-phase protein and a general marker of several pathological processes, including infection, tissue damage, cancer, and chronic inflammatory disease [1]. Low levels of CRP can be measured accurately, and it is thus possible to identify individuals with low-grade inflammation (LGI), defined as CRP measurement above 3 mg/L but below 10 mg/L [2,3]. LGI is associated with increased risk of several diseases [4]: e.g., coronary heart disease [5,6], rheumatoid arthritis [7], and cancer [8]. Also, LGI is widely used in cardiovascular risk assessment [9,10].

Health-Related Quality of Life (HRQL) represent individuals’ subjective assessment of their mental and physical well-being. HRQL is accepted as a health indicator in health surveys, and is one of the strongest predictors of survival in the general population [11]. Further, HRQL is used to measure disease burden [12] and as a screening tool for specific mental diseases [13]. HRQL changes with age, and most studies agree that around 30% of the variance in self-rated health can be explained by genetic factors [14]. Chronic diseases, such as hypertension, diabetes, chronic obstructive pulmonary disease (COPD), and heart disease are associated with impaired HRQL, especially physical health [15]. HRQL is also associated with future events (e.g., heart disease), where the risk of illness increases with decreasing HRQL [16].

LGI is suggested as one factor linking HRQL with future health outcomes [17–19]. Although previous studies conducted in the general population indicate a robust relation between higher levels of proinflammatory cytokines (IL-6 and TNF-α) [17,18,20–22], acute-phase proteins (CRP and fibrinogen) [21,23–25] or erythrocyte sedimentation rate [26] and HRQL, the lack of sex-stratification in most studies could conceal sex-specific effects [18,21,22,24], and sufficient adjustment was not performed in all studies. Furthermore, the mechanisms underlying the association of HRQL with morbidity and mortality risk are poorly understood. It is of interest to investigate whether higher levels of acute-phase proteins have an independent association with HRQL to tease out whether higher levels of inflammatory markers are rather a mediator of a confounder.

Therefore, we examined the association between HRQL, LGI (expressed as elevated CRP), and objectively measured clinical indicators among healthy individuals without clinical symptoms using a mediation analysis model. We hypothesized that LGI correlates with a lower physical and mental HRQL score in a population of blood donors, which has not previously been investigated in this context.

## Materials and methods

### Study design and population

We studied participants from The Danish Blood Donor Study (DBDS), which has been described in detail previously [27–30]. Briefly, DBDS was initiated March 2010 as a multicentre, public-health study and biobank (www.dbds.dk). Currently, more than 110,000 blood donors aged 18–67 years participate in DBDS. At study enrolment, participants completed a four-page questionnaire on health-related items (HRQL), including current smoking status, alcohol consumption, physical activity, diet, anthropometric measurements, and (among women) use of combined oral contraception (OC), childbirth, and menopausal status. Plasma samples were stored, and donors gave permission to link their data to public registers. Overall, fewer than 5% of invited donors have declined to participate [30]. Implicitly, all participants in the present study were healthy, non-pregnant, and non-lactating adults testing negative for HIV, hepatitis B, and C.

According to the definition of LGI, 176 individuals with CRP levels above 10 mg/L were excluded. Likewise, 138 individuals were excluded on the basis of missing values on CRP. For the SF-12 up to two missing items can be accepted and replaced with the population mean [31]. Nevertheless, we decided to exclude participants with more than one missing value in the SF-12 questionnaire, and a mean value (adjusted for age and sex) was used as a substitute for those with one missing item. A total of 919 participants were thus excluded. In the primary analysis, we excluded those who failed to respond to certain questionnaire items, namely: current smoking (1,024 participants were excluded), height (1,017), weight (927), body mass index (BMI) (1,017), physical activity at work (942), leisure-time physical activity (946), meat intake (956), fish intake (1,008) and childbirth (575). Five postmenopausal women were excluded as they stated usage of combined oral contraception. For waist circumference, 1,985 responses were missing, and these participants were excluded from analyses in which waist circumference was used as predictor. In total, 1,413 women and 1,102 men were thus excluded.

### Measurements: C-reactive protein

From 1 March to 31 December 2010, 25,877 participants were included in DBDS. High-sensitivity CRP was measured in plasma samples drawn at enrolment from 17,024 participants. CRP was measured by a commercially available, high-sensitivity assay on an automated system (Ortho Vitros 5600, Ortho Clinical Diagnostics, Rochester, NY, USA). Blood was collected in ethylenediamine tetraacetic acid (EDTA)-containing, gel-separated tubes, centrifuged within six hours and stored at -20°C. Samples were stored for less than 12 months before CRP measurement. Measurements on thawed EDTA plasma were validated against fresh serum, which is the test material recommended by the manufacturer. The two measures were highly correlated (R^2^: 0.98). The CRP levels were somewhat lower in plasma (slope: 0.935). The coefficient of variation for the assay was below 4.8%. The measuring range of the assay was 0.10 to 15.00 mg/L. A default value of 0.05 mg/L was assigned to samples below the lower limit of detection; no samples beyond the upper limit of detection were encountered.

### Measurements: Health-Related Quality of Life (HRQL)

The 36-item short-form health survey (SF-36) is the most commonly used descriptive HRQL measure and was developed for population surveys [32,33]. The 12-item short-form (SF-12) is an abbreviated version of SF-36. The questionnaire completed by all participants at enrolment included the SF-12 version one [32,33], see S1 File. SF-12 consists of 12 items from the SF-36, and the result is reported as a physical (PCS) and mental (MCS) component summary score. PCS and MCS are computed using scores of twelve questions and range from 0 to 100, where 0 score indicates the lowest and 100 indicates the highest level of health. As recommended by Quality Metric Inc., the SF-12 was scored giving weights to the individual items and adding the sum to a constant (57.65693 and 60.58847) in PCS and MCS, respectively. In Denmark, the median PCS and MCS are 56 and 54, respectively [32]. The correlation between SF-12 and SF-36 in Denmark on PCS and MCS is 0.95 and 0.96, respectively [34,35]. SF-36 and SF-12 are valid for assessing the general health in populations, and both scores are used as screening tools for specific diseases.

### Definition of low-grade inflammation

We used the common practice of defining LGI as CRP level above 3 mg/L and below or equal to 10 mg/L [2,27,36]. Recent study has suggested participant-specific thresholds may be necessary when using one time measurement of CRP to distinguish acute from chronic inflammation in obese women to avoid introducing bias into study findings [37]. Therefore, sensitivity analyses of participants with CRP level above 10 mg/L are performed.

### Statistical analysis

The characterization of the study population was stratified by sex, and data were presented as numbers, means with ranges, or frequency statistics. Groups were compared by the two-sample t-test for normally distributed data, the X^2^ test for categorical data, and the Mann-Whitney U-test for non-normally distributed data.

The relationship between primary outcomes (PCS or MCS) and predictors was explored by mediation analysis with sensitivity analyses to examine whether LGI is a mediator in the association between predictors and outcomes [38] adjusted for smoking, age, BMI, waist circumference, physical activity at work and in leisure stratified as detailed below (e.g., the association between BMI and PCS was explored with LGI as possible mediator adjusted for smoking, age, waist circumference, physical activity at work and in leisure). Furthermore, unadjusted and adjusted multivariable linear regression analysis were conducted to compare with mediation analysis model.

Interaction tests between LGI and the predictors were included in all models. Analyses were stratified by the use of OC among premenopausal women, as reported in the questionnaire and confirmed in the Danish National Prescription Register, because OC is a strong predictor of LGI [39]. Residuals were plotted against predicted values to assess model assumptions of normality and independence.

The explored predictors included LGI (yes/no), defined as CRP above 3 mg/L and below or equal to 10 mg/L [2,3]; BMI as a continuous variable; waist circumference as a continuous variable; current smoking (yes/no), cumulative tobacco consumption in pack years (one pack year was defined as 20 g of tobacco every day for one year [40], where a cigarette was estimated to contain 1 g of tobacco; and a cheroot, a cigar, or a pipe 3 g of tobacco [41]); age as a continuous variable; physical activity at work (high vs. low, see definition below); leisure-time physical activity (high vs. low, see definition below)[42,43], childbirth (yes/no); meat intake (yes/no), defined as meat consumption or vegetarian; fish intake (yes/no), defined as consumption of fish more than twice weekly.

Low physical activity at work was defined as mainly sitting or standing and occasionally walking. High physical activity at work was defined as walking and lifting occasionally; or heavy physical work. Low leisure-time physical activity was defined as light physical activity less than 2 hours per week or two to four times per week. High leisure-time physical activity was defined as light physical activity more than 4 hours a week, or fatiguing physical activity for at least 2 hours per week [44].

SF-12 MCS and PCS follow the Poisson distribution in Danish populations [32]. Transformation of data by a power of four and five could approximate the normal distribution for MCS and PCS, respectively [45]. Because the transformed data are more difficult to interpret, we performed and presented all calculations on the original scale. The correlations were later checked by performing the regression analysis after transformation. Cronbach’s α was estimated to evaluate internal consistency of the SF-12.

Multiple imputation was used to remedy missing data in predictor variables that were assumed to be missing at random. Only relevant predictor variables for outcomes were included in the imputation procedure (BMI, current smoking status, waist circumference, physical activity at work and leisure-time) based on complete observed data variables of age and stratified by sex. A total of 100 imputed datasets were created and used for imputation modelling. Sensitivity analyses were performed to assess robustness [46,47].

Results were presented as regression coefficients (RC) with 95% confidence intervals (CI). Statistical analyses were performed using Stata/MP 14.1 for Windows (StataCorp LP, College Station, Texas, USA). A *p*-value below 0.05 was considered significant.

### Ethics statement

Oral and written informed consent was obtained from all participants. The study was approved by The Scientific Ethical Committee of Central Denmark (M-20090237). Additionally, the biobank and research database have been approved by the Danish Data Protection Agency (2007-58-0015).

## Results

### Characteristics of the study population

The characteristics of the study population are presented in Table 1 and have previously been reported [39]. Briefly, the study comprised 17,024 individuals (47.3% women). The median age was 37.8 years for women and 40.1 years for men. The median PCS score was 55.9 and 56.0, and MCS score was 53.5 and 54.8, for women and men, respectively. Among women and men 17.1% and 15.8% were smokers, respectively. In the cohort, a total of 1,542 (10.3%) had LGI.

**Table 1:** Characteristics of case-complete participants stratified by sex (n = 15,380).

	Women	Men
**Numbers of participants**	7,249 (47.3)	8,131 (52.7)
**Age, years**	37.8 (27.3; 47.8)	40.1 (30.3; 49.7)
**Body mass index (BMI), kg/m^2^**	23.5 (21.5; 26.1)	25.1 (23.3; 27.4)
**Obesity, BMI≥30 kg/m^2^**	673 (9.3)	806 (9.9)
**Current smoker**	1,228 (17.1)	1,292 (15.8)
**Waist circumference (cm)**	82.0 (76.0; 90.0)	92.0 (86.0; 99.0)
**PCS (point)**	55.9 (53.9; 57.3)	56.0 (53.9; 57.2)
**MCS (point)**	53.5 (50.1; 56.7)	54.8 (51.6; 56.8)
**C-reactive protein, mg/L**	0.67 (0.19; 1.90)	0.42 (0.11; 1.09)
Low-grade inflammation (LGI)*	1,056 (14.6)	486 (6.0)
**C-reactive protein > 5 mg/L**	440 (6.1)	177 (2.2)
**Combined oral contraception**	2,143 (29.6)	-

Numbers (%) or medians (interquartile ranges).

PCS: physical component score. MCS: mental component score.

* LGI defined as 3 mg/L<CRP≤10 mg/L. Note this group also includes participants with CRP greater than 5 mg/L.

Characteristics of the group with missing responses are presented in Table A in S2 File. Compared with the study population, this group had a higher fraction of women, a higher prevalence of obesity and current smokers, lower PCS and MCS scores for both sexes, higher CRP levels, and fewer using OC among women.

Characterisation of participants with CRP levels above 10 mg/L are presented in Table B in S2 File. Analysis including these individuals did not change results, see Table C in S2 File.

### Predictors of PCS

PCS and associations with LGI among women using OC, women not using OC, and men are presented in Tables 2–5. An interaction between LGI and OC (p = 0.009) was observed as described previously [27] and consequently analyses were stratified according to the use of OC. No interaction between LGI and age in any strata was observed. The mediation analyses showed a statistically significant, however only a small, effect of smoking on PCS is mediated through LGI only among women not using OC and men (the average mediated effect was 3.0% among women not using OC and 4.8% among men, see Table 2). In the age-only adjusted linear regression analysis, LGI was associated with lower PCS in all three donor strata (Table 3). Effects persisted, although attenuated, after further adjusting for current smoking, BMI, waist circumference, physical activity at work and leisure-time (women using OC: RC = -0.36 points lower PCS in participants with LGI vs no LGI, CI: -0.94 to -0.19; women not using OC: RC = -0.63, CI: -1.05 to -0.21 and men: RC = -0.76, CI: -1.10 to -0.42), see Table 4. Estimates by multiple imputation were similar to results of the case-complete analyses, see Table 5.

**Table 2:** Mediation analysis of predictors for physical component score with LGI as mediator on case-complete participants with adjustments (n = 14,371).

	Women using OC (n = 1,984)		Women not using OC (n = 4,770)		Men (n = 7,617)	
Outcome PCS / Mediator LGI	RC	95% CI	RC	95% CI	RC	95% CI
**BMI (kg/m^2^)**						
ACME	-0.002	-0.006; -0.0002	-0.0005	-0.001; 0.0001	-0.0003	-0.0006; 0.0001
Direct effect	-0.057	-0.12; -0.01	-0.086	-0.13; -0.04	-0.11	-0.15; -0.06
Total effect	-0.059	-0.12; -0.01	-0.086	-0.13; -0.04	-0.11	-0.15; -0.06
Proportion of total effect (%)	3.3	-2.3; 18.6	0.5	0.4; 1.1	0.2	0.1; 0.4
**Current smoker (yes/no)**						
ACME	0.006	-0.02; 0.03	-0.02	-0.05; -0.003	-0.03	-0.05; -0.01
Direct effect	-0.65	-1.10; -0.18	-0.64	-0.92; -0.36	-0.56	-0.77; -0.35
Total effect	-0.64	-1.09; -0.17	-0.66	-0.94; -0.37	-0.59	-0.80; -0.37
Proportion of total effect (%)	-1.0	-3.4; -0.6	3.0	2.1; 5.3	4.8	3.5; 7.6
**Age (10 year increment)**						
ACME	-0.01	-0.03; -0.002	0.001	-0.001; 0.006	-0.004	-0.006; -0.001
Direct effect	0.14	-0.08; 0.36	-0.27	-0.33; -0.20	-0.18	-0.26; -0.10
Total effect	0.13	-0.09; 0.36	-0.26	-0.34; -0.19	-0.18	-0.26; -0.10
Proportion of total effect (%)	-6.3	-98.3; 80.7	-0.5	-0.7; -0.4	2.0	1.4; 3.6
**Waist (10 cm increment)**						
ACME	-0.01	-0.03; 0.002	-0.004	-0.01; 0.0001	0.005	-5.57·10^-18; 0.001
Direct effect	-0.46	-0.61; -0.30	-0.38	-0.48; -0.28	-0.42	-0.52; -0.32
Total effect	-0.47	-0.62; -0.31	-0.39	-0.48; -0.28	-0.42	-0.52; -0.31
Proportion of total effect (%)	2.2	1.7; 3.3	1.0	0.8; 1.4	-1.2	-1.7; -1.0
**Physical activity, work (low/high)**						
ACME	-0.01	-0.03; -0.06	-0.01	-0.03; 0.002	-0.01	-0.02; 0.005
Direct effect	-0.11	-0.50; 0.30	-0.42	-0.67; -0.16	-0.53	-0.72; -0.33
Total effect	-0.12	-0.50; 0.30	-0.43	-0.68; -0.17	-0.54	-0.72; -0.33
Proportion of total effect (%)	2.1	-48.8; 60.0	2.1	1.3; 5.2	1.1	0.8; 1.7
**Physical activity, leisure (low/high)**						
ACME	0.01	-0.0002; 0.04	0.01	0.02; 0.03	0.01	-0.02; 0.02
Direct effect	0.48	0.16; 0.82	0.74	0.52; 0.97	0.70	0.57; 0.84
Total effect	0.49	0.18; 0.84	0.75	0.54; 0.99	0.71	0.58; 0.85
Proportion of total effect (%)	2.8	1.7; 7.9	1.7	1.3; 2.4	0.9	0.8; 1.1

RC: regression coefficient, OC: combined oral contraception, PCS: physical component score, MCS: mental component score, CRP: C-reactive protein, BMI: body mass index, LGI: low-grade inflammation (defined as 3 mg/L < CRP ≤ 10 mg/L), CI: confidence interval

Note: Analysis performed without missing responses in waist circumference variable

**Table 3:** Predictors of PCS and MCS estimated by linear regression analysis adjusted for age of case-complete participants (n = 15,380).

	Women using OC (n = 2,143)		Women not using OC (n = 5,106)		Men (n = 8,131)		Men and women not using OC (n = 13,237)	
**Outcome: PCS**	**RC**	**(95% CI)**	**RC**	**(95% CI)**	**RC**	**(95% CI)**	**RC**	**(95% CI)**
Low-grade inflammation (LGI)	-0.77	(-1.10; -0.44)	-1.54	(-1.95; -1.14)	-1.32	(-1.67; -0.98)	-1.53	(-1.94; -1.16)
Age (10 year increment)	-0.04	(-0.25; 0.18)	-0.35	(-0.44; -0.26)	-0.29	(-0.36; -0.23)	-0.32	(-0.37; -0.26)
Female							0.04	(-0.18; 0.10)
Female x LGI							0.23	(-0.30; 0.76)
**Outcome: MCS**	**RC**	**(95% CI)**	**RC**	**(95% CI)**	**RC**	**(95% CI)**	**RC**	**(95% CI)**
Low-grade inflammation (LGI)	-0.04	(-0.66; 0.57)	-0.16	(-0.80; 0.48)	0.17	(-0.70; 0.6)	-0.13	(-0.74; 0.47)
Age (10 year increment)	0.99	(0.59; 1.39)	1.23	(1.08; 1.37)	0.99	(0.88; 1.09)	1.07	(0.99; 1.16)
Female							1.14	(0.92; 1.36)
Female x LGI							-0.07	(-0.88; 0.75)

RC: regression coefficient, OC: combined oral contraception, PCS: physical component score, MCS: mental component score, CRP: C-reactive protein, LGI defined as 3 mg/L < CRP ≤ 10 mg/L, CI: confidence interval.

**Table 4:** Predictors of PCS and MCS estimated by multivariable linear regression analysis of case-complete participants (n = 14,371).

	Women using OC (n = 1,984)		Women not using OC (n = 4,770)		Men (n = 7,617)		Men and women not using OC (n = 12,387)	
**Outcome: PCS**	**RC**	**(95% CI)**	**RC**	**(95% CI)**	**RC**	**(95% CI)**	**RC**	**(95% CI)**
Low-grade inflammation (LGI)	-0.36	(-0.94; -0.19)	-0.63	(-1.05; -0.21)	-0.76	(-1.10; -0.42)	-0.78	(-1.13; -0.44)
BMI (kg/m^2^)	-0.06	(-0.13; -0.01)	-0.09	(-0.13; -0.04)	-0.11	(-0.14; -0.07)	-0.10	(-0.12; -0.07)
Current smoker (yes/no)	-0.67	(-1.09; -0.25)	-0.67	(-0.95; -0.38)	-0.57	(-0.80; -0.36)	-0.62	(-0.79; -0.44)
Age (10 year increment)	0.14	(-0.07; 0.36)	-0.26	(-0.36; -0.17)	-0.16	(-0.23; -0.09)	-0.20	(-0.26; -0.15)
Waist (10 cm increment)	-0.39	(-0.62; -0.17)	-0.25	(-0.41; -0.09)	-0.30	(-0.41; -0.19)	-0.29	(-0.38; -0.19)
Physical activity, work (low/high)	-0.11	(-0.49; 0.27)	-0.43	(-0.69; -0.17)	-0.51	(-0.70; -0.33)	-0.48	(-0.64; -0.33)
Physical activity, leisure (low/high)	0.48	(0.14; 0.81)	0.73	(0.50; 0.96)	0.70	(0.52; 0.87)	0.71	(0.57; 0.85)
Female							-0.18	(-0.48; 0.12)
Female x LGI							-0.02	(-0.54; 0.49)
**Outcome: MCS**	**RC**	**(95% CI)**	**RC**	**(95% CI)**	**RC**	**(95% CI)**	**RC**	**(95% CI)**
Low-grade inflammation (LGI)	0.27	(-0.37; 0.92)	-0.05	(-0.73; 0.62)	0.04	(-0.49; 0.57)	0.12	(-0.50; 0.74)
BMI (kg/m^2^)	-0.005	(-0.13; 0.12)	0.05	(-0.02; 0.12)	-0.03	(-0.08; 0.03)	0.01	(-0.04; 0.05)
Current smoker (yes/no)	-1.05	(-1.83; -0.26)	-0.89	(-1.35; -0.43)	-0.82	(-1.16; -0.47)	-0.84	(-1.12; -0.57)
Age (10 year increment)	1.17	(0.76; 1.58)	1.24	(1.09; 1.39)	1.00	(0.90; 1.11)	1.08	(1.01; 1.17)
Waist (10 cm increment)	-0.40	(-0.82; 0.019)	-0.12	(-0.37; 0.13)	-0.03	(-0.21; 0.15)	-0.06	(-0.21; -0.08)
Physical activity, work (low/high)	-0.08	(-0.79; 0.62)	0.47	(-0.05; 0.90)	0.64	(0.35; 0.93)	0.56	(0.32; 0.80)
Physical activity, leisure (low/high)	0.99	(0.36; 1.61)	1.04	(0.68; 1.42)	1.16	(0.88; 1.44)	1.11	(0.89; 1.34)
Female							1.01	(0.79; 1.24)
Female x LGI							-0.12	(-0.94; 0.69)


RC: regression coefficient, OC: combined oral contraception, PCS: physical component score, MCS: mental component score, CRP: C-reactive protein, BMI: body mass index, LGI defined as 3 mg/L < CRP ≤ 10 mg/L, CI: confidence interval.

Note: Analysis performed without missing responses in abdominal obesity variable

**Table 5:** Predictors of PCS and MCS estimated by multiple imputation of multivariable linear regression analysis (n = 17,024).

	Women using OC (n = 2,249)		Women not using OC (n = 5,984)		Men (n = 8,791)		Men and women not using OC (n = 14,775)	
**Outcome: PCS**	**RC**	**(95% CI)**	**RC**	**(95% CI)**	**RC**	**(95% CI)**	**RC**	**(95% CI)**
Low-grade inflammation (LGI)	-0.37	(-0.72; -0.03)	-0.81	(-1.22;-0.39)	-0.80	(-1.14;-0.47)	-0.74	(-1.12;-0.35)
BMI (kg/m^2^)	-0.06	(-0.13; -0.01)	-0.08	(-0.12;-0.003)	-0.11	(-0.14;-0.07)	-0.09	(-0.12;-0.06)
Current smoker (yes/no)	-0.71	(-1.13; -0.29)	-0.66	(-0.95;-0.37)	-0.60	(-0.82;-0.38)	-0.62	(-0.80;-0.45)
Age (10 year increment)	0.12	(-0.10; 0.35)	-0.29	(-0.38;-0.20)	-0.16	(-0.23;-0.10)	-0.21	(-0.27;-0.16)
Waist (10 cm increment)	-0.39	(-0.61; -0.17)	-0.27	(-0.42;-0.11)	-0.29	(-0.40;-0.18)	-0.29	(-0.37;-0.20)
Physical activity, work (low/high)	-0.18	(-0.61; 0.18)	-0.41	(-0.67;-0.15)	-0.53	(-0.72;-0.35)	-0.49	(-0.64;-0.34)
Physical activity, leisure (low/high)	0.49	(0.16; 0.83)	0.77	(0.53;1.00)	0.70	(0.53;0.88)	0.73	(0.59;0.87)
Female							-0.26	(-0.39;-0.13)
Female x LGI							-0.19	(-0.71; 0.25)
**Outcome: MCS**	**RC**	**(95% CI)**	**RC**	**(95% CI)**	**RC**	**(95% CI)**	**RC**	**(95% CI)**
Low-grade inflammation (LGI)	0.25	(-0.40;0.89)	-0.15	(-0.81;0.51)	0.001	(-0.52;0.53)	0.04	(-0.57;0.65)
BMI (kg/m^2^)	-0.004	(-0.13;0.12)	0.06	(-0.01;0.12)	-0.03	(-0.08;0.03)	0.01	(-0.03;0.05)
Current smoker (yes/no)	-1.12	(-1.90;-0.34)	-0.89	(-1.34;-0.44)	-0.84	(-1.18;-0.50)	-0.86	(-1.13;-0.59)
Age (10 year increment)	1.10	(0.68;1.52)	1.20	(1.05;1.34)	0.97	(0.87;1.08)	1.05	(0.97;1.14)
Waist (10 cm increment)	-0.36	(-0.77;0.006)	-0.09	(-0.34;0.16)	-0.09	(-0.18;0.17)	-0.04	(-0.18;0.10)
Physical activity, work (low/high)	-0.13	(-0.83;0.57)	0.47	(0.06;0.88)	0.61	(0.32;0.90)	0.54	(0.30;0.77)
Physical activity, leisure (low/high)	1.05	(0.43;1.67)	1.04	(0.68;1.41)	1.15	(0.88;1.43)	1.08	(0.85;1.31)
Female							1.05	(0.84;1.27)
Female x LGI							-0.12	(-0.91;0.53)

RC: regression coefficient, OC: combined oral contraception, PCS: physical component score, MCS: mental component score, CRP: C-reactive protein, BMI: body mass index, LGI defined as 3 mg/L < CRP ≤ 10 mg/L, CI: confidence interval.

* Note: Analysis performed without missing responses in abdominal obesity variable.

BMI, current smoking, and waist circumference were negatively associated with PCS in all three donor strata. Further, increasing age and high physical activity at work were also negatively associated with PCS, but only among women not using OC, and among men. In contrast, high physical activity in leisure-time increased PCS in all three donor strata (Table 2 and 4). Estimates by multiple imputation were also similar to the effects of case-complete analyses, see Table 5.

### Predictors of MCS

LGI was not associated with MCS in any strata and the effect of predictors was not mediated by LGI (Table 6). Both increasing age and physical activity in leisure-time significantly increased MCS in all strata, whereas physical activity at work increased MCS among men and women not using OC. Current smoking status was the only strong and statistically significant predictor of a reduction in MCS in all strata (Tables 4 and 6). Estimates by multiple imputation demonstrated the same trend as results from the case-complete analyses (Table 5).

**Table 6:** Mediation analysis of predictors for mental component score with LGI as mediator on case-complete participants with adjustments (n = 14,371).

	Women using OC (n = 1,984)		Women not using OC (n = 4,770)		Men (n = 7,617)	
Outcome MCS / Mediator LGI	RC	95% CI	RC	95% CI	RC	95% CI
**BMI (kg/m^2^)**						
ACME	0.001	-0.001; 0.006	-0.00003	-0.0006; 0.0007	0.00001	-0.0002; 0.0002
Direct effect	0.001	-0.14; 0.15	0.05	-0.001; 0.10	-0.03	-0.07; 0.02
Total effect	0.003	-0.14; 0.15	0.05	-0.002; 0.10	-0.03	-0.07; 0.02
Proportion of total effect (%)	0.9	-24.4; 37.9	-0.006	-0.4; 0.2	-0.04	-0.6; 0.4
**Current smoker (yes/no)**						
ACME	-0.05	-0.03; 0.01	-0.003	-0.03; 0.02	0.001	-0.02; 0.02
Direct effect	-1.01	-1.72; -0.28	-0.87	-1.34; -0.39	-0.80	-1.16; -0.43
Total effect	-1.02	-1.72; -0.29	-0.88	-1.34; -0.40	-0.80	-1.16; -0.43
Proportion of total effect (%)	0.5	0.3; 1.6	0.3	0.2; 0.8	-0.001	-0.1; -0.05
**Age (10 year increment)**						
ACME	0.01	-0.01; 0.03	0.0001	-0.002; 0.03	0.0001	-0.003; 0.03
Direct effect	1.18	0.81; 1.56	1.25	1.12; 1.39	1.00	0.91; 1.10
Total effect	1.19	0.82; 1.57	1.25	1.12; 1.39	1.00	0.91; 1.10
Proportion of total effect (%)	0.6	0.5; 0.9	0.01	0.013; 0.02	0.01	0.013; 0.02
**Waist (10 cm increment)**						
ACME	0.0007	-0.0007; 0.002	-0.0005	-0.006; 0.004	-0.000009	-0.005; 0.005
Direct effect	-0.35	-0.67; -0.02	-0.08	-0.28; 0.13	-0.08	-0.23; 0.08
Total effect	-0.34	-0.66; -0.01	-0.08	-0.28; 0.13	-0.08	-0.23; 0.08
Proportion of total effect (%)	-2.0	-10.2; 0.9	0.4	-7.5; 8.2	0.1	-1.0; 1.0
**Physical activity, work (low/high)**						
ACME	0.004	-0.01; 0.03	-0.001	-0.01; 0.01	0.0001	-0.01; 0.01
Direct effect	-0.06	-0.63; 0.54	0.49	0.10; 0.90	0.64	0.35; 0.94
Total effect	-0.06	-0.63; 0.54	0.49	0.10; 0.90	0.64	0.35; 0.94
Proportion of total effect (%)	-0.9	-20.6; 18.3	-0.2	-1.1; -0.1	0.02	0.01; 0.03
**Physical activity, leisure (low/high)**						
ACME	-0.01	-0.04; 0.01	0.002	-0.01; 0.02	-0.0001	-0.01; 0.01
Direct effect	1.00	0.42; 1.61	1.06	0.67; 1.47	1.17	0.91; 1.45
Total effect	0.99	0.42; 1.59	1.06	0.68; 1.47	1.17	0.91; 1.45
Proportion of total effect (%)	-1.0	-2.4; -0.6	0.2	0.1; 0.3	-0.01	-0.01; -0.008

RC: regression coefficient, OC: combined oral contraception, PCS: physical component score, MCS: mental component score, CRP: C-reactive protein, BMI: body mass index, LGI: low-grade inflammation (defined as 3 mg/L < CRP ≤ 10 mg/L), CI: confidence interval

Note: Analysis performed without missing responses in waist circumference variable

### Diet and childbirths had no impact on HRQL

The relation between PCS and MCS with childbirth, meat intake, and fish intake were statistically insignificant in univariate regression analysis, therefore not included in further analysis, see Table D in S2 File.

### Transformation of MCS and PCS

The results for the transformed PCS and MCS were nearly identical to the results for untransformed PCS and MCS (Table E in S2 File). Cronbach’s α was 0.77 and 0.75 for PCS and MCS, respectively, which is acceptable.

## Discussion

In the current study, LGI was associated with a reduction of PCS among women and men but had no impact on MCS. A significant but small proportion of the effect by smoking on PCS is mediated through LGI. BMI and current smoking also had a negative effect on PCS. Physical activity in leisure-time was the only predictor of an increased PCS. In both sexes, current smoking was negatively associated with MCS whereas aging and physical activity in leisure-time were associated with an increased MCS.

### LGI affects negatively on PCS but had no impact on MCS

To the best of our knowledge, the present study is the first to combine HRQL subjective health assessment with an inflammatory biomarker and objectively measured clinical indicators in a mediation analysis model, demonstrating that among relevant predictors of HRQL only a partial effect of smoking on self-reported physical health is mediated through LGI.

Our finding in this large study strengthens the growing evidence of an association between low self-rated health and higher levels of inflammatory markers, including IL-6 [17,18,21,22], TNF-α [19,20], CRP [21,23–25], erythrocyte sedimentation rate (ESR) [26], and fibrinogen [48]. Our study consists of a large population of healthy blood donors of a wide age range using the comprehensive HRQL as the tool for the assessment of subjective health and analysis stratified for sex, and use of OC. Several studies have shown similar association between CRP and lower score in HRQL, but only among women [23], others only among men [24], or in a design not stratified by sex [21]. Conversely, one study reported no relation between low HRQL and CRP, but only with higher levels of IL-6 [18]. Compared with the current study, previous studies differ in terms of the inflammation marker investigated, the assessment method of self-rated health, size of study population, and the adjustments included in the analysis of the association between HRQL and LGI. The hitherto largest study, which includes 43,110 men aged 18–21 years only, reports that elevated levels of ESR are associated with an increased risk of low HRQL score by 7.7% after adjustments [26]. Among studies including both sexes and a large sample size, low HRQL is related to higher levels of CRP only among women in an Asian population although a lower CRP threshold was defined as LGI (hsCRP above 1 mg/L) [23]. By contrast, another study including 13,236 participants found similar associations only among men; however, the age range was 24–34 [24] compared with 40–69 years [23]. Our reported negative association of LGI on physical health among both genders is in concordance with one previous study with 13,773 apparently healthy individuals from a routine health examination at a medical centre demonstrating higher levels of CRP among the self-rated health ‘average’ group compared to ‘excellent’, however, individuals reporting ‘very poor’ and ‘poor’ were not included [25].

### Relation between lifestyle factors and HRQL

The current study was able to replicate the independent negative relation between PCS and lifestyle factors with respect to current smoking, physical activity, and obesity in a cohort of healthy individuals. These results are in agreement with previous studies that report a similar association with low HRQL among participants selected by sex, age, and region in the population [49,50]. Unlike previous studies, we find that the negative impact of smoking on PCS is partly mediated through LGI, however, the percentage of the effect mediated through LGI is marginal.

Further, we found no association between consumption of meat or fish and HRQL; a finding that agrees well with previous study which fails to demonstrate any association between HRQL and Mediterranean Diet Score (low-dietary-quality vs. high-dietary-quality)[50]. Waist circumference or rather abdominal obesity as a predictor of LGI has been described [27]; however, to our knowledge no other studies have shown abdominal obesity as a predictor of reduced PCS or low HRQL. Contrary to previous studies, we investigated the effects of physical activity at work and in leisure-time separately with findings in accordance with recent studies with regards to the effect of physical activity in leisure-time [26,49,50]. Physical activity at work reduced PCS among men and women not using OC; whereas the same trend among women using OC was statistically insignificant. The lack of significance could be explained by lack of statistical power in the latter stratum.

Recent results from a study including 1,546 adults aged 21–100 years suggest surprising observation that aging is associated with improved mental health among older adults, despite loss of physical and cognitive function [51]. In our study, we did indeed find that aging improved MCS and reduced PCS among women not using OC, and among men. The lack of a significant relation between aging and reduction of PCS among women using OC could possibly be explained by a lower and more narrow age group (median age is 26.5 years among women using OC and 38.4 years among women not using OC [27]) which is related to the age dependent difference of choice of contraception among women. Various explanations for an improved mental health in later life have been proposed, including increased wisdom and experience with aging, or a suggestion that older individuals tend to be more emotionally balanced, better at complex social decision-making, and tend to process information with a more positive attitude. Such ballast in life and a generally “positive outlook” could explain higher level of subjective well-being in later life [52].

### Strengths and limitations of the study

Our study comprised a large and homogeneous population of healthy individuals, in as much as because blood donors must comply with strict criteria to be allowed to donate and are permanently excluded from blood donation if diagnosed with certain chronic diseases, including diabetes, cancer, hypertension, or even hypercholesterolemia. Because blood donors are a selected group, being generally healthier than the background population, they are uniquely suitable for studies of health associations among individuals without clinical symptoms. In addition, the age range of our study population was broad (18–67 years), a more balanced sex distribution compared to previous studies [23,25], and our use of HRQL with the SF-12 questionnaire as descriptive measure of self-rated health rather than single-item measure [17–19,23,24,26] gives more information regarding perceived physical and mental health. Further, participants with more than one missing value in the SF-12 items were excluded, even though up to two missing values is generally acceptable in studies of this kind. Moreover, our study included not only a mediation analyses but also multivariable linear regression analyses that essentially report similar effects; however, the contribution of the mediation analyses gives further understanding of whether other predictors lead to LGI which then affects HRQL and to which extent the effect of predictors on physical and mental HRQL is mediated through LGI. Our finding indicates that LGI had an independent impact on HRQL scores, although a partial and small effect of smoking on PCS is mediated through LGI.

The mediation analyses and linear regression analysis was supplemented with multiple imputation modelling in the analysis strategy, which is an accepted and recommended procedure for handling missing data and to obtain more accurate and unbiased effect estimates [46,47]. In the current study, the group with missing values overall did not match completely with the study population with complete data in terms of distribution of sex and other predictors, as mentioned earlier. Multiple imputation did not alter direction of effects. In addition, our statistical analysis approach prompted inclusion of relevant predictors in adjusting for confounders. Our study is limited by relying on CRP as the only biomarker of low-grade inflammation, where additional, simultaneous biomarkers would be preferable. Finally, the cross-sectional design precludes any conclusion regarding causal relationships.

## Conclusion

LGI was an independent predictor of lower self-rated physical health score in HRQL in both men and women but was not associated with MCS. BMI, current smoking status, age, waist circumference, and physical activity were predictors of the physical and mental health component in HRQL. Only a small effect of smoking on PCS was mediated through LGI.

## Supporting information

S1 File Danish and English questionnaires for the Danish Blood Donor Study. (PDF)

S2 File **Table A. Missing group (n = 2,515).** Numbers (%) or medians (interquartile ranges) of participants in the particular predictor variable with values available. BMI: body mass index, PCS: physical component score, MCS: mental component score, LGI defined as 3 mg/L < CRP ≤ 10 mg/L, CRP: C-reactive protein. * Number of participants with missing values in the particular predictor variable. **Table B. Excluded participants with C-reactive protein levels above 10 mg/L (n = 176).** Numbers (%) or medians (interquartile ranges). **Table C. Sensitivity analysis, predictors of PCS and MCS estimated by multivariable linear regression analysis of case-complete participants with C-reactive protein level > 10 mg/L included (n = 14,547).** RC: regression coefficient, OC: oral contraception, PCS: physical component score, MCS: mental component score, CRP: C-reactive protein, BMI: body mass index, LGI: low-grade inflammation. Note: Analysis performed without missing responses in abdominal obesity variable. **Table D. Diet and childbirth as predictors of PCS and MCS estimated by univariate linear regression analysis of case-complete participants (n = 15,380).** RC: regression coefficient, OC: oral contraception, PCS: physical component score, MCS: mental component score, CI: confidence interval. * Meat consumption defined as meat intake or vegetarian. ** Fish consumption defined as fish intake more than twice weekly (yes) or not (no). **Table E. Predictors of PCS and MCS estimated by multivariable linear regression analysis after transformation of data by power of four (MCS) and five (PCS).** RC: regression coefficient, OC: oral contraception, PCS: physical component score, MCS: mental component score, CRP: C-reactive protein, BMI: body mass index, LGI defined as 3 mg/L < CRP ≤ 10 mg/L.
(PDF)
