# Correction: Low-grade inflammation is negatively associated with physical Health-Related Quality of Life in healthy individuals: Results from The Danish Blood Donor Study (DBDS)

**DOI:** 10.1371/journal.pone.0216339

**Published:** 2019-05-28

**Authors:** The PLOS ONE Staff

The Funding statement is incorrect. The publisher apologizes for the error. The correct Funding statement is as follows: The Danish Blood Donor Study was funded by: The Danish Council for Independent Research - Medical Sciences (http://ufm.dk/en/research-and-innovation/councils-and-commissions/the-danish-council-for-independent-research, grant number: 09-069412); The Danish Administrative Regions (http://www.regioner.dk); Bloddonorernes Forskningsfond (https://forening.bloddonor.dk/om-bloddonorerne-idanmark/fonde); A.P. Møller Foundation for the Advancement of Medical Science (https://www.apmollerfonde.dk/ansoegning/laegefonden.aspx); The Danish Bio and Genome Bank (http://www.regioner.dk/rbgben).

The first author was funded by Aarhus University (http://phd.health.au.dk/application/opencalls/), Health Research Fund of Central Denmark Region (https://www.rm.dk/sundhed/faginfo/forskning/region-midtjyllands-sundhedsvidenskabelige-forskningsfond/), Aase & Ejnar Danielsens Fond (https://danielsensfond.dk), The Højmosegård Grant, and A.P. Møller Foundation for the Advancement of Medical Science. None of the funders had any influence on study design, data collection and analysis, decision to publish, or preparation of the manuscript.
