# Supplementary material for: Low-grade inflammation is negatively associated with physical Health-Related Quality of Life in healthy individuals: Results from The Danish Blood Donor Study (DBDS)
Source: PLoS One. 2019 Mar 28;14(3):e0214468. doi: 10.1371/journal.pone.0214468 (PMC6438577; doi:10.1371/journal.pone.0214468)
Supplement: S1 File — (PDF) [file pone.0214468.s001.pdf]

Identification number

THE DANISH BLOOD DONOR STUDY

We will kindly ask you to answer some questions regarding your health and lifestyle. If there are any questions you cannot answer, just skip them.
The questions in the survey can be answered by ticking a box or by writing a number. Please fill the survey with a black or blue pen, as the answers will be read by a machine. Do not use a pencil. Numbers should be easy to interpret, shown in the example below:

0 1 2 3 4 5 6 7 8 9

If you write a wrong number in a field, you should correct it like this: 19
If you tick in a wrong box you must cross out the wrong box until it is completely filled. Then you can tick the right box.

Please contact the staff if you have any questions about the survey.
Please return the completed questionnaire to the staff.

Thank you in advance for your help!

The following questions are about your perception of your health

1. How do you think your health is in general? (Tick one box only)

Excellent	Very good	Good	Fair	Poor
☐	☐	☐	☐	☐

2. The following questions are about activities of everyday life. Does your health limit you in these activities? If so, how much?

	Yes, limited a lot	Yes, limited a little	No, not limited at all
Moderate activities, such as moving a table, pushing a vacuum cleaner or cycling ……….	☐	☐	☐
Climbing several flights of stairs………………	☐	☐	☐

3. During the past 4 weeks have you had any of the following problems with your work or other regular activities as a result of <u>your physical health</u>?

	Yes	No
Accomplished less than you would like …………………………………..	☐	☐
Were limited in the kind of work or other activities.……………………..	☐	☐

4. During the past 4 weeks, were you limited in the kind of work you do or other regular activities as a result of any <u>emotional problems (such as feeling depressed or anxious)</u>?

	Yes	No
Accomplished less than you would like …………………………………..	☐	☐
Did not do work or other activities as carefully as usual…………………	☐	☐

5. During the past 4 weeks, how much did pain interfere with your normal work (including both work outside the home and housework)? (*Tick one box only*)

Not at all	A little bit	Moderately	Quite a bit	Extremely
☐	☐	☐	☐	☐

6. The next three questions are about how you feel and how things have been during the past 4 weeks. How much of the time during the past 4 weeks…

	All of the time	Most of the time	A good bit of the time	Some of the time	A little of the time	None of the time
… have you felt calm and paeceful?	☐	☐	☐	☐	☐	☐
… did you have a lot of energy?…..	☐	☐	☐	☐	☐	☐
… have you felt downhearted and blue?...	☐	☐	☐	☐	☐	☐

7. During the past 4 weeks, how much of the time has your physical health or emotional problems interfered with your social activities (like visiting with wriends, relatives, etc.)?

All of the time	Most of the time	Some of the time	A little of the time	None of the time
☐	☐	☐	☐	☐

	Better	Same	Poorer
8. How do you consider your health compared to other on your own age?.............................	☐	☐	☐

<u>The next question is about general infectious disease</u>

	Yes	No
9. Have you ever been told from a doctor that you have kissing disease (mononucleosis)? …………………………………………………………..	☐	☐

If yes: How old was you then?..…. Age: |__|__|

<u>The next questions are about vitamin supplements, iron supplements, diet and alcohol</u>

10. During the past 4 weeks, have you been taking multivitamins?

- Yes, everyday..…………….. …………………………………… ☐
- Yes, 4-6 times per week……………………………………….. ☐
- Yes, 1-3 times per week …… …………………...…………. ☐
- Yes, less than 3 times within the last 4 weeks……………….. ☐
- No……...………………………………………………………. ☐

11. During the past 4 weeks, have you been taking iron supplements?

- Yes, everyday..…………….. …………………………………… ☐
- Yes, 4-6 times per week……………………………………….. ☐
- Yes, 1-3 times per week …… …………………...…………. ☐
- Yes, less than 3 times within the last 4 weeks……………….. ☐
- No……...………………………………………………………. ☐

	Yes	No
12. Last time you donated blood, did you get any iron supplements?..…….	☐	☐

If yes: How many iron tablets did you take?..................................... Times: |__|__|

13. How many times in 1 week do you eat meat? ……………………… Times: |__|__|

14. How many times in 1 week do you eat fish?...……………………… Times: |__|__|

15. How many eggs do you eat in 1 week? …………………………….. Number: |__|__|

16. How often do you drink …

	Never / almost never	Few times a month	Few times a week	Everyday / almost everyday	Average number **per week**
milk? ……………..	☐	☐	☐	☐	Glasses: |__|__|
coffee? …………..	☐	☐	☐	☐	Cups: |__|__|
tea?......................	☐	☐	☐	☐	Cups: |__|__|
beer?....................	☐	☐	☐	☐	Bottles: |__|__|
wine?....................	☐	☐	☐	☐	Glasses: |__|__|
spirits? ……………	☐	☐	☐	☐	Units: |__|__|

The next questions are about smoking

17. Do you smoke?

Yes, more than 1 cigarette / cerut / pipe per day…..………. ☐ **Go to question 20**

Yes, but less than 1 cigarette / cerut / pipe per day……….. ☐ **Go to question 20**

No ……………………………………………………………… ☐

18. Have you smoked before?

Yes, more than 1 cigarette / cheroot / pipe per day…..……. ☐

Yes, but less than 1 cigarette / cheroot / pipe per day……… ☐

No ……………………………………………………………… ☐ **Go to question 23**

19. When did you stop smoking? ………………………………………. Year: |__|__|

20. How many years did you smoke regularly? ……………………… Number of whole years: |__|__|

21. How much do you smoke (or did) during 1 day in average?

Number of cigarettes……………………………………………................. |__|__|

Number of cheroots or cigars..…………………………………………..... |__|__|

Number of pipes…………………………………………….……………. |__|__|

22. Do you use nicotine replacement (gum, plaster or similar)? ……….. Yes ☐ No ☐

If yes: How long time have you used it?................................. Number of whole years: |__|__|

23. How many hours per day are you exposed for passive smoking? …… Hours: |__|__|

24. Was there smoking in your childhood home?..……………………………. Yes ☐ No ☐

Only for women – men please go to question 30

25. How old was you, when you got your period?.............……………….. Age: |__|__|

26. Are you in menopause?...........…………………………………………… Yes ☐ No ☐

If no: How many days do your period last? Number of days: |__|__|

Yes No

If yes: Did it stop because of medication or other treatment?............ ☐ ☐

How old was you, when your period stopped?...................... Age: |__|__|

Yes No

27. Have you given birth? .... ☐ ☐

28. Have you given breast-feed?.. ☐ ☐

If yes: In total, how long how you been breast-feeding?...... Number of months |__|__|

Yes No

29. Do you use birth control? .... ☐ ☐

If yes: mark the type of birth control you use now:

- P-piller .... ☐
- Mini-piller .... ☐
- Almindelig spiral ... ☐
- Hormonspiral .... ☐
- P-stav .... ☐
- P-ring eller p-plaster ☐
- Anden prævention ☐

The next questions are about exercise, weight and height

30. How would you describe your work or daily occupation? *(*tick one box only)

☞ Also completed by homemakers, students and unemployed.

- Mostly sitting........... .... ☐
- Sitting or standing, occasionally walking........................... ☐
- Walking, occasionally lifting.. ☐
- Heavy physical work.. ☐

---

31. How would you describe your spare time? *(*tick one box only)

☞ *Include transportation to and from work/school.*

- Light physical activity less than 2 hours per week .... ☐
- Light physical activity 2-4 hours per week................................ ☐
- Light physical activity more than 4 hours per week..................... ☐
- Strenuous physical activity for 2-4 hours a week .... ☐
- Strenuous physical activity more than 4 hours per week or regular hard exercise.......... .... ☐

---

32. What is your weight right now? .... lp: |__|__|__|

---

33. What was your weight 5 years ago (about)?.................................. lp: |__|__|__|

---

34. What is the most you have ever weighed?.................................. lp: |__|__|__|

☞ *Except of pregnancy.*

---

35. What is your waist circumference measured from your navel?........... in: |__|__|__|

---

36. How tall are you (without shoes)?... in: |__|__|__|

Thank you very much for your help

Please return the completed questionnaire to the staff

DET DANSKE BLODDONORSTUDIE

Til tappenummer

Vi vil bede dig besvare nogle spørgsmål, der først og fremmest drejer sig om dit helbred og livsstil. Hvis der er spørgsmål du ikke kan svare på, kan du springe dem over.

Spørgsmålene i skemaet kan besvares ved, at du sætter kryds i en rubrik eller ved at skrive tal. Da svarene bliver indlæst maskinelt vil vi bede dig udfylde skemaet med blå eller sort kuglepen. Brug ikke blyant. Tal skal være nemme at tolke, hvilket er vist i nedenstående eksempel:

0 1 2 3 4 5 6 7 8 9

Har du skrevet et forkert tal i et felt, skal du rette det på følgende måde: 19

Hvis du kommer til at sætte kryds i en forkert rubrik, skal du overstrege det forkerte kryds til rubrikken er helt fyldt ud. Herefter kan du sætte kryds i den rigtige rubrik.

Hvis du har spørgsmål til undersøgelsen, kan du spørge personalet på tappestedet.

Skemaet bedes afleveret til blodbankens personale, når du er færdig med at udfylde det.

På forhånd tak for hjælpen!

De følgende spørgsmål handler om din opfattelse af dit helbred

1. Hvordan synes du dit helbred er alt i alt? (*Sæt kun et kryds*)

Fremragende	Vældig godt	Godt	Mindre godt	Dårligt
☐	☐	☐	☐	☐

2. De følgende spørgsmål handler om aktiviteter i dagligdagen. Er du på grund af dit helbred begrænset i disse aktiviteter? I så fald hvor meget?

	Ja, meget begrænset	Ja, lidt begrænset	Nej, slet ikke begrænset
Lettere aktivieteter, såsom at flytte et bord, støvsuge eller cykle …………………………	☐	☐	☐
At gå flere etager op ad trapper………………	☐	☐	☐

3. Har du indenfor de sidste 4 uger haft nogen af følgende problemer med dit arbejde eller andre daglige aktiviteter på grund af dit fysiske helbred?

	Ja	Nej
Jeg har nået mindre, end jeg gerne ville …………………………………..	☐	☐
Jeg har været begrænset i hvilken slags arbejde eller andre aktiviteter, jeg har kunnet udføre ………………………………………………………..	☐	☐

4. Har du inden for de sidste 4 uger haft nogen af følgende problemer med dit arbejde eller andre daglige aktiviteter på grund af følelsesmæssige problemer?

	Ja	Nej
Jeg har nået mindre, end jeg gerne ville …………………………………..	☐	☐
Jeg har udført mit arbejde eller andre aktiviteter mindre omhyggeligt, end jeg plejer ………………………………………………………………	☐	☐

5. Inden for de sidste 4 uger hvor meget har fysisk smerte vanskeliggjort dit daglige arbejde (både arbejde udenfor hjemmet og husarbejde)? (*Sæt kun et kryds*)

Slet ikke	Lidt	Noget	En hel del	Virkeligt meget
☐	☐	☐	☐	☐

Ikke til print

6. Disse spørgsmål handler om, hvordan du har haft det i de sidste 4 uger. Hvor stor en del af tiden de sidste 4 uger…

	Hele tiden	Det meste af tiden	En hel del af tiden	Noget af tiden	Lidt af tiden	På intet tids-punkt
… har du følt dig rolig og afslappet?	☐	☐	☐	☐	☐	☐
… har du været fuld af energi? …….	☐	☐	☐	☐	☐	☐
… har du følt dig trist til mode? …….	☐	☐	☐	☐	☐	☐

7. Inden for de sidste 4 uger, hvor stor en del af tiden har dit fysiske helbred eller følelsesmæssige problemer gjort det vanskeligt at se andre mennesker (f.eks. besøge venner, slægtninge osv.)?

Hele tiden	Det meste af tiden	Noget af tiden	Lidt af tiden	På intet tidspunkt
☐	☐	☐	☐	☐

	Bedre	Det samme	Dårligere
8. Hvordan synes du dit helbred er, sammenlignet med andre på din alder?.........	☐	☐	☐

Det følgende spørgsmål handler om almindelige infektioner

	Ja	Nej
9. Har du nogen sinde fået at vide af en læge, at du har haft kyssesyge (mononukleose)? …………………………………………………………..	☐	☐

Hvis ja: Hvor gammel var du dengang?... Alder: |__|__| år

Følgende spørgsmål handler om vitamintilskud, jerntilskud, kost og alkohol

10. Har du taget multivitaminpiller indenfor de se sidste 4 uger?

Ja, dagligt ………………….. …………………………………..	☐
Ja, 4-6 gange pr. uge ……… …………………………………..	☐
Ja, 1-3 gange pr. uge ……… …………………………………..	☐
Ja, mindre end 3 gange indenfor de sidste 4 uger …………..	☐
Nej, aldrig ………………………………………………………..	☐

11. Har du taget jerntabletter indenfor de sidste 4 uger?

Ja, dagligt ………………….. …………………………………..	☐
Ja, 4-6 gange pr. uge ……… …………………………………..	☐
Ja, 1-3 gange pr. uge ……… …………………………………..	☐
Ja, mindre end 3 gange indenfor de sidste 4 uger …………..	☐
Nej, aldrig ………………………………………………………..	☐

	Ja	Nej
12. Fik du udleveret jerntabletter sidste gang du gav blod? ………………….	☐	☐

Hvis ja: Hvor mange jerntabletter tog du?... Antal: |__|__|

13. Hvor mange gange om ugen spiser du kød? ………………………. Antal gange: |__|__|

14. Hvor mange gange om ugen spiser du fisk? ………………………. Antal gange: |__|__|

15. Hvor mange æg spiser du om ugen? ……………………………….. Antal æg: |__|__|

ikke til print

16. Hvor ofte drikker du …

	Aldrig / næsten aldrig	Nogle gange om måneden	Nogle gange om ugen	Dagligt / næsten dagligt	Antal i gennemsnit **pr. uge**
mælk? ……………..	☐	☐	☐	☐	Glas: |__|__|
kaffe? ……………..	☐	☐	☐	☐	Kopper: |__|__|
te? …………………	☐	☐	☐	☐	Kopper: |__|__|
øl? …………………	☐	☐	☐	☐	Flasker: |__|__|
vin? …………………	☐	☐	☐	☐	Glas: |__|__|
spiritus? ……………	☐	☐	☐	☐	Genstande: |__|__|

De følgende spørgsmål handler om rygning

17. Ryger du nu?

Ja, mere end 1 cigaret / cerut / pibestop om dagen ……… ☐ **Gå til sp. 20**

Ja, men mindre end 1 cigaret / cerut / pibestop om dagen ☐ **Gå til sp. 20**

Nej ………………………………………………………………. ☐

18. Har du røget tidligere?

Ja, mere end 1 cigaret / cerut / pibestop om dagen ……..… ☐

Ja, men mindre end 1 cigaret / cerut / pibestop om dagen ☐

Nej ………………………………………………………………. ☐ **Gå til sp. 23**

19. Hvornår holdt du op med at ryge? …………………………………. Årstal: |__|__|

20. Hvor mange år har du røget regelmæssigt? …………………….. Antal hele år: |__|__|

21. Hvor meget ryger du (eller røg) du i gennemsnit om dagen?

Antal cigaretter ……..……………………………………………….................. |__|__|

Antal cerutter eller cigarer.……………………………………………………... |__|__|

Antal pibestop ……………………………………………………..…………… |__|__|

22. Bruger du nikotinsubstitut (tyggegummi, plaster eller lignende)? ……….. Ja ☐ Nej ☐

Hvis ja: Hvor længe har du brugt det? .... Antal hele år: |__|__|

23. Hvor mange timer pr dag bliver du udsat for passiv rygning? …………… Timer: |__|__|

24. Blev der røget i dit barndomshjem? ………………………………………… Ja ☐ Nej ☐

Kun for kvinder – mænd bedes gå til spørgsmål 30

25. Hvor gammel var du, da dine menstruationer begyndte?………………….. Alder: |__|__| år

26. Er dine menstruationer holdt op? …………………………………………… Ja ☐ Nej ☐

Hvis nej: Hvor mange dage varer dine menstruationer vanligvis? Antal dage: |__|__|

Hvis ja: Ophørte de pga. medicin eller anden behandling? Ja ☐ Nej ☐

Hvor gammel var du, da dine menstruationer holdt op?......... Alder: |__|__| år

Ikke til print

		Ja	Nej
27.	Har du født børn? …………………………………………………………..	☐	☐
28.	Har du ammet? ………………………………………………………………..	☐	☐

Hvis ja: Hvor længe har du sammenlagt ammet dine børn? Antal måneder: |__|__|

		Ja	Nej
29.	Bruger du prævention? ……………………………………………………	☐	☐

Hvis ja: marker den prævention du bruger for

- P-piller …………… ☐
- Mini-piller ………… ☐
- Almindelig spiral … ☐
- Hormonspiral ……. ☐
- P-stav ……………. ☐
- P-ring eller p-plaster ☐
- Anden prævention ☐

De følgende spørgsmål handler om motion, vægt og højde

30. Hvordan vil du beskrive dit arbejde eller din daglige beskæftigelse? *(sæt kun et kryds)*

☞ Udfyldes også af hjemmegående, studerende og arbejdsløse.

- Overvejende siddende ………………………………………….. ☐
- Siddende eller stående, af og til gående ……………………….. ☐
- Gående, af og til løft ………………………………………… ☐
- Tungt fysisk arbejde ………………………………………… ☐

31. Hvordan vil du beskrive din fritid? *(sæt kun et kryds)*

☞ *Medregn også transport til og fra arbejde/skole.*

- Let fysisk aktivitet mindre end 2 timer om ugen ………………… ☐
- Let fysisk aktivitet i 2-4 timer om ugen ……………………………. ☐
- Let fysisk aktivitet mere end 4 timer om ugen …………………… ☐
- Anstrengende fysisk aktivitet i 2-4 timer om ugen ……………… ☐
- Anstrengende fysisk aktivitet i mere end 4 timer om ugen eller regelmæssig hård træning ………………………………………. ☐

32. Hvad vejer du nu? ……………………………………………………… kg: |__|__|__|

33. Hvad vejede du for 5 år siden (cirka)?…………………………………… kg: |__|__|__|

34. Hvad er det meste du nogensinde har vejet?………………………….. kg: |__|__|__|

☞ *Bortset fra under graviditet.*

35. Hvad er dit taljemål eller livvidde målt ud for navlen? ………………… cm: |__|__|__|

36. Hvor høj er du (uden sko)? ………………………………………………… cm: |__|__|__|

Mange tak for din hjælp

Sørgeskemaet bedes afleveret på tappestedet

ikke til print
