# Supplementary material for: Low-grade inflammation is negatively associated with physical Health-Related Quality of Life in healthy individuals: Results from The Danish Blood Donor Study (DBDS)
Source: PLoS One. 2019 Mar 28;14(3):e0214468. doi: 10.1371/journal.pone.0214468 (PMC6438577; doi:10.1371/journal.pone.0214468)
Supplement: S2 File — Table A. Missing group (n = 2,515). Numbers (%) or medians (interquartile ranges) of participants in the particular predictor variable with values available. BMI: body mass index, PCS: physical component score, MCS: mental component score, LGI defined as 3 mg/L < CRP ≤ 10 mg/L, CRP: C-reactive protein. * Number of participants with missing values in the particular predictor variable. Table B. Excluded participants with C-reactive protein levels above 10 mg/L (n = 176). Numbers (%) or medians (interquartile ranges). Table C. Sensitivity analysis, predictors of PCS and MCS estimated by multivariable linear regression analysis of case-complete participants with C-reactive protein level > 10 mg/L included (n = 14,547). RC: regression coefficient, OC: oral contraception, PCS: physical component score, MCS: mental component score, CRP: C-reactive protein, BMI: body mass index, LGI: low-grade inflammation. Note: Analysis performed without missing responses in abdominal obesity variable. Table D. Diet and childbirth as predictors of PCS and MCS estimated by univariate linear regression analysis of case-complete participants (n = 15,380). RC: regression coefficient, OC: oral contraception, PCS: physical component score, MCS: mental component score, CI: confidence interval. * Meat consumption defined as meat intake or vegetarian. ** Fish consumption defined as fish intake more than twice weekly (yes) or not (no). Table E. Predictors of PCS and MCS estimated by multivariable linear regression analysis after transformation of data by power of four (MCS) and five (PCS). RC: regression coefficient, OC: oral contraception, PCS: physical component score, MCS: mental component score, CRP: C-reactive protein, BMI: body mass index, LGI defined as 3 mg/L < CRP ≤ 10 mg/L. (PDF) [file pone.0214468.s002.pdf]

S2 Supporting Information File

PLOS ONE

Low-grade inflammation is negatively associated with physical Health Related Quality of Life in healthy individuals: results from The Danish Blood Donor Study (DBDS)

S2 Table A. Missing group (n=2,515)

	Women	Men	No. of missing response*
**Numbers of participants**	1,413 (56.2)	1,102 (43.8)	
**Age, years**	37.9 (26.5; 48.4)	39.2 (30.1; 50.6)	
**BMI, kg/m^2**	24.4 (21.9; 28.2)	25.2 (23.4; 27.8)	1,017
**Obesity, BMI≥30 kg/m^2**	155 (19.2)	115 (15.0)	1,017
**Current smoker**	166 (22.0)	133 (18.1)	1,024
**Waist circumference (cm)**	85.0 (78.0; 92.0)	94.0 (88.0; 100.0)	1,985
**PCS (point)**	54.5 (51.4; 56.2)	55.0 (54.1; 56.2)	0
**MCS (point)**	51.8 (47.4; 54.8)	54.0 (52.0; 56.7)	0
**CRP, mg/L**	0.86 (0.27; 2.35)	0.50 (0.15; 1.26)	0
**Low-grade inflammation (LGI)***	252 (17.8)	81 (7.35)	0
**CRP > 5 mg/L**	100 (7.1)	33 (3.0)	0
**Combined oral contraception**	243(17.2)	-	

S2 Table B. Excluded participants with C-reactive protein levels above 10 mg/L (n=176).

	**Women**	**Men**
**Numbers of participants**	135 (76.7)	41 (23.3)
**Age, years**	31.6 (25.5; 42.5)	43.1 (32.6; 53.9)
**Body mass index (BMI), kg/m^2**	28.3 (25.3; 33.1)	26.9 (24.2; 30.2)
**Obesity, BMI≥30 kg/m^2**	52 (38.5)	11 (26.8)
**Current smoker**	37 (27.4)	9 (22.0)
**Waist circumference (cm)**	91.5 (82.0; 102.0)	97.0 (89.0; 105.0)
**Physical Component Score, PCS (point)**	55.5 (53.2;56.8)	54.7 (51.2; 56.6)
**Mental Component Score, MCS (point)**	53.2 (49.0; 56.7)	54.8 (49.9; 56.7)
**C-reactive protein, CRP (mg/L)**	12.1 (11.0; 13.4)	11.5 (10.6; 12.9)
**Combined oral contraception**	83 (61.5)	-

S2 Table C. Sensitivity analysis, predictors of PCS and MCS estimated by multivariable linear regression analysis of case-complete participants with C-reactive protein level > 10 mg/L included (n=14,547).

	Women using OC (n=2,064)		Women not using OC (n=4,824)		Men (n=7,659)		Men and women not using OC (n=12,483)	
**Outcome PCS**	**RC**	**(95% CI)**	**RC**	**(95% CI)**	**RC**	**(95% CI)**	**RC**	**(95% CI)**
Low-grade inflammation (LGI)	-0.38	(-0.74; -0.04)	-0.68	(-1.10; -0.26)	-0.79	(-1.13; -0.45)	-0.78	(-1.17; -0.39)
BMI (kg/m^2)	-0.11	(-0.16; -0.05)	-1.13	(-0.16; -0.09)	-0.14	(-0.17; -0.10)	-1.20	(-1.43; -0.98)
Current smoker (yes/no)	-0.69	(-1.11; -0.26)	-0.69	(-0.97; -0.40)	-0.58	(-0.81; -0.36)	-0.61	(-0.79; -0.44)
Age (10 year increment)	0.13	(-0.09; 0.35)	-0.27	(-0.36; -0.18)	-0.16	(-0.23; -0.09)	-0.22	(-0.28; -0.17)
Waist (10 cm increment)	-0.40	(-0.76; -0.05)	-0.24	(-0.51; -0.03)	-0.47	(-0.67; -0.27)	-0.70	(-0.84; -0.56)
Physical activity, work (low/high)	-0.12	(-0.50; 0.26)	-0.43	(-0.70; -0.17)	-0.50	(-0.69; -0.32)	-0.49	(-0.65; -0.34)
Physical activity, leisure (low/high)	0.50	(0.17; 0.84)	0.75	(0.52; 0.99)	0.70	(0.52; 0.88)	0.73	(0.59; 0.87)
Female							-0.18	(-0.32; -0.04)
Female x LGI							-0.02	(-0.54; 0.49)
**Outcome MCS**	**RC**	**(95% CI)**	**RC**	**(95% CI)**	**RC**	**(95% CI)**	**RC**	**(95% CI)**
Low-grade inflammation (LGI)	0.24	(-0.40; 0.89)	-0.10	(-0.77; 0.58)	0.03	(-0.50; 0.57)	0.07	(-0.55; 0.68)
BMI (kg/m^2)	-0.06	(-0.16; 0.04)	0.04	(-0.01; 0.10)	-0.02	(-0.07; 0.03)	0.16	(-0.20; 0.52)
Current smoker (yes/no)	-1.06	(-1.84; -0.27)	-0.89	(-1.35; -0.44)	-0.82	(-1.16; -0.47)	-0.85	(-1.13; -0.57)
Age (10 year increment)	1.15	(0.74; 1.56)	1.25	(1.10; 1.40)	1.00	(0.90; 1.11)	1.10	(1.01; 1.18)
Waist (10 cm increment)	-0.34	(-0.99; 0.32)	-0.39	(-0.84; 0.04)	-0.18	(-0.21;0.15)	-0.27	(-0.50; -0.04)
Physical activity, work (low/high)	-0.09	(-0.80; 0.61)	0.47	(0.05; 0.90)	0.64	(0.34; 0.93)	0.56	(0.32; 0.80)
Physical activity, leisure (low/high)	1.02	(0.39; 1.64)	1.04	(0.67; 1.41)	1.15	(0.87; 1.42)	1.10	(0.88; 1.32)
Female							1.01	(0.79; 1.24)
Female x LGI							-0.12	(-0.94; 0.69)

S2 Table D. Diet and childbirth as predictors of PCS and MCS estimated by univariate linear regression analysis of case-complete participants (n=15,380)

	Women using OC (n=2,143)		Women not using OC (n=5,106)		Men (n=8,131)	
**Outcome: PCS**	**RC**	**(95% CI)**	**RC**	**(95% CI)**	**RC**	**(95% CI)**
Meat consumption (yes/no) *	-0.02	(-1.26; 1.31)	-0.84	(-1.74; 0.46)	-1.05	(-2.30; 0.20)
Fish consumption (yes/no) **	0.34	(-0.04; 0.66)	0.16	(-0.06; 0.39)	0.30	(-0.13; 0.47)
Childbirth (yes/no)	-0.31	(-0.68; 0.05)	-0.35	(-0.60; 0.12)	-	-
**Outcome: MCS**	**RC**	**(95% CI)**	**RC**	**(95% CI)**	**RC**	**(95% CI)**
Meat consumption (yes/no)	2.07	(-0.30; 4.44)	2.27	(-0.83; 3.70)	1.76	(-0.19; 3.70)
Fish consumption (yes/no)	0.66	(-0.10; 1.23)	0.01	(-0.35; 0.36)	0.08	(-0.18; 0.34)
Childbirth (yes/no)	0.89	(-0.21; 1.56)	1.47	(0.01; 1.85)	-	-

S2 Table E. Predictors of PCS and MCS estimated by multivariable linear regression analysis after transformation of data by power of four (MCS) and five(PCS)

	Women using OC (n=1,984)		Women not using OC (n=4,770)		Men (n=7,617)	
**Outcome PCS**	**RC**	**P**	**RC**	**P**	**RC**	**P**
Low-grade inflammation (LGI)	↓	0.001	↓	<0.001	↓	<0.001
BMI (kg/m^2)	↓	0.004	↓	<0.001	↓	<0.001
Current smoker (yes/no)	↓	0.031	↓	<0.001	↓	<0.001
Age (10 year increment)	↑	0.39	↓	<0.001	↓	<0.001
Waist (10 cm increment)	↓	<0.001	↓	<0.001	↓	<0.001
Physical activity, work (low/high)	↓	0.59	↓	0.017	↓	<0.001
Physical activity, leisure (low/high)	↑	0.002	↑	<0.001	↑	<0.001
**Outcome MCS**	**RC**	**P**	**RC**	**P**	**RC**	**P**
Low-grade inflammation (LGI)	↑	0.28	↓	0.72	↑	0.35
BMI (kg/m^2)	↓	0.30	↑	0.056	↓	0.63
Current smoker (yes/no)	↓	0.018	↓	0.016	↓	<0.001
Age (10 year increment)	↑	<0.001	↑	<0.001	↑	<0.001
Waist (10 cm increment)	↓	0.082	↓	0.087	↓	0.12
Physical activity, work (low/high)	↑	0.39	↑	0.071	↑	<0.001
Physical activity, leisure (low/high)	↑	0.003	↑	<0.001	↑	<0.001
